# Global Meta-Analysis on the Association between Behcet Syndrome and Polymorphisms from the HLA Class I (A, B, and C) and Class II (DRB1, DQB1, and DPB1) Genes

**DOI:** 10.1155/2021/9348697

**Published:** 2021-12-13

**Authors:** Cristina Capittini, Chiara Rebuffi, Marco Vincenzo Lenti, Antonio Di Sabatino, Carmine Tinelli, Miryam Martinetti, Annalisa De Silvestri

**Affiliations:** ^1^Scientific Direction, IRCCS Policlinico San Matteo Foundation, Pavia, Italy; ^2^IRCCS Istituto Giannina Gaslini, Scientific Direction, Italy; ^3^First Department of Internal Medicine, San Matteo Hospital Foundation, University of Pavia, Pavia, Italy; ^4^University of Pavia, Italy

## Abstract

Behcet syndrome (BS) is a multisystemic perivasculitis whose genetic susceptibility is linked to HLA region. We first meta-analysed all HLA class I and II genes involved in BS susceptibility in all ethnic groups worldwide. We identified 1141 articles and finally included 31 case-control studies after multiple rounds of selection. We analysed frequencies for 24 HLA-A alleles (3 alleles for HLA-A∗26 at four digits), 50 HLA-B alleles (11 alleles for HLA-B∗51 at four digits), 15 HLA-C alleles, 16 HLA-DRB1 alleles, 6 HLA-DQB1 alleles, and 15 HLA-DPB1 alleles. We meta-analysed only HLA allelic frequencies from at least three studies; therefore, we investigated 21 alleles out of 140. Going from 7.00 to 1.6 OR, we found 11 class I alleles conferring risk for BS: B∗51 : 08, B∗51, B∗51 : 01, B∗51 : 02, DQB1∗03, A∗26 : 01, Cw∗14, Cw∗15, Cw∗16, B∗15, and A∗26. Overall, the studies included populations from Europe (Greece, Spain, Italy, Germany, and Ireland), Asia (Korea, China, China Han, and Thailand), Middle East (Israel, Saudi Arabia, and Iran), and Morocco (as no other North-African population was included). We collected a number of ethnical groups sufficient to conduct an ethnic-specific meta-analysis where Europeans showed 11.25 OR for B∗51:08 and Japan 3.50 OR for A∗26 : 01. A remarkable result was that the most frequent HLA − B∗51 two-digit alleles associated with BS were different among populations: HLA − B∗51 : 08 in Europe, HLA − B∗51 : 01 in Turkey, and HLA − B∗51 : 02 in Japan. Overall, we discussed our real-world results with other imputation studies.

## 1. Introduction

Behcet syndrome (BS) is an autoinflammatory multisystemic neutrophilic perivasculitis characterized by recurrent inflammatory flares causing protean clinical manifestations [1].

Although the diagnosis is based only on clinical signs set by the International Criteria for Behçet's Disease (ICBD), from a genetic point of view, the disease has been historically linked first to human leukocyte antigen- (HLA-) B5 serotype, then to the HLA-B51 molecule/allele [2].

The geographic distribution of BS over the centuries shows a connection with specific populations, in particular those ones settled along the Silk Road, which is a network of trade routes connecting East Asia with Southern Europe passing through Middle East lands and East Africa [3].

The HLA region shows an extensive variation in the number of both genes and alleles. HLA genes are the most polymorphic ones in the human genome, and more than 8450 alleles have been discovered at the HLA-B locus. It is worldwide accepted that the extensive polymorphism of the HLA region is the result of selective pressures driven by the functional role of HLA molecules in the immune response. In fact, the highest degree of polymorphism is toward the peptide-binding region [4].

From 2009 onwards, seven genome-wide association studies (GWAS) in BS have been published. Overall, the following subjects were analysed in worldwide GWAS: 2576 Turkish BS with 2728 healthy controls; 975 Japanese BS with 1013 healthy controls; 336 Western Europeans, Middle Eastern, and Turkish BS with 5843 healthy ethnically matched controls; 379 Korean BS with 800 healthy controls; and 703 Chinese BS with 2110 healthy controls [5–11].

Fei and colleagues performed the first genome-wide association study in a cohort of 152 Turkish BS patients and 172 healthy ethnically matched controls [5]. They identified genetic associations between BS and the following single-nucleotide polymorphisms (SNPs): rs317711 (7p15-p14) in CPVL (carboxypeptidase vitellogenic-like, expressed on human macrophages and trimming peptides for antigen presentation); rs4936742 (11q24) in UBASH3B (ubiquitin-associated and SH3 domain containing B, promotes accumulation of T-cell receptors and EGFR on the cell surface); rs9513584 (13q32) in UBAC2 (ubiquitin-associated domain (UBA) containing 2, negatively regulates the canonical Wnt signaling pathway in the lymphocytes); and rs2061634 (9q22) in KIAA1529 and rs11206377 (1p34) in LOC100129342 that both do not have known function up to now [5]. Overall, although identifying novel candidate SNPs, they did not find the disease-causing polymorphisms.

Remmers and colleagues performed a GWAS with 311,459 SNPs in 1215 Turkish BS and 1278 controls. They confirmed the known association of BS with the HLA-B∗51 alleles and identified an association at rs1518111 (Intron Variant chr1:206771300) IL10 (interleukin-10, controls cytokine activity and the inflammatory response of macrophages). The rs1518111 A allele was associated with diminished mRNA expression and low protein production [6].

Mizuki and colleagues conducted a GWAS in a Japanese cohort of 612 BS patients and 740 controls. They identified two associations: rs12119179 (1p31.3) in IL23R-IL12RB2 and rs1554286 (1q32.1) in IL10 [7].

Hou and colleagues enrolled 149 Chinese BS patients and 951 controls in the initial GWAS and 554 patients and 1159 controls in the replication study. They identified that the susceptibility SNPs rs7574070, rs7572482, and rs897200 around 2q32.2-q32.3 were maps STAT4 (signal transducer and activator of transcription 4, involved in IL12 signaling). Carriers of rs897200 risk genotype AA showed increased expression of STAT4 and increased levels of IL17 messenger RNA and protein. Mainly, the clinical disease severity score was higher in carriers of the rs897200 risk genotype AA [8].

Lee and colleagues performed a GWAS in 379 Korean BD and 800 controls. A replication study was performed in 363 BD Japanese and 272 controls. They found a novel association of BD with SNIPs located in the GIMAP (GTPase IMAP family) cluster (7q36.1): the rs1608157 in a minor allele dominant model and the rs11769828 allele based. Furthermore, using a fine mapping study, they also identified an association with rs1522596 in GIMAP4 (GTPase IMAP family member 4), rs10266069 and rs10256482 in GIMAP2 (GTPase IMAP family member 2), and rs2286900 in GIMAP1 (GTPase IMAP family member 1). Overall, their results suggest that the GIMAP cluster may be involved in BS, even though without any verified connection [9].

Kirino and colleagues performed a GWAS of 779,465 SNPs with imputed genotypes in 1209 Turkish BS individuals and 1278 controls. They identified associations at CCR1 (C-C chemokine receptor type 1) (3p21.31), STAT4, and KLRC4 (NKG2-F type II integral membrane protein, a receptor for the recognition of MHC class I HLA-E molecules by NK cells) (12p13.2). They also found two SNPs in ERAP1 (endoplasmic reticulum aminopeptidase 1 that trims peptide for the generation of most HLA class I-binding peptides) (5q15). They also found evidence for interaction between HLA-B∗51 and ERAP1 [10].

Kappen and colleagues performed a GWAS on 336 Turkish, Western Europeans, and Middle Eastern BD cases and 5843 multiethnic birth cohort (from the Netherlands), using linear regression models corrected for population stratification. They identified SNPs mapping to the HLA region (6p21.33) [11].

Overall, all these GWAS showed a limited number of novel locus associations. In fact, also using the most advanced molecular techniques, the HLA region still remains the most involved in BS susceptibility, mostly in Turkish patients.

Another issue concerns the unequal distribution of BS among different ethnic groups, and the use of GWAS and bioinformatics tools in cohorts of mixed ethnicity does not seem to be an effective solution.

This is the first meta-analysis that considers all HLA class I and II genes involved in BS susceptibility in all ethnic groups worldwide.

## 2. Materials and Methods

This study followed the PRISMA guidelines [12].

### 2.1. Protocol

We drafted a protocol including: review question, eligibility criteria, primary and secondary endpoints, search strategy, methods for data extraction, study quality assessment, risk of bias assessment, strategy for data synthesis, and statistical methodology.

On April 29th 2019, the protocol entitled “Association between HLA class I (A, B, and C) and class II (DRB1, DQB1, and DPB1) polymorphisms and Behcet Syndrome: a meta-analysis” was published in the PROSPERO International prospective register of systematic reviews (http://www.crd.york.ac.uk/PROSPEROCRD42019130390).

### 2.2. Search Strategy

We performed a systematic search in PubMed, Embase, Web of Science, and Scopus databases, retrieving all publications (case-control, cross-sectional, and retrospective cohort studies or mixed design like nested case-control and cohort studies) on the association between HLA class I and II alleles and Behcet Syndrome (BS) in adult patients (>18 years).

We searched all English, Italian, Spanish, French, and Turkish-written articles published in up to December 2020. An expert librarian performed the search using the following MeSh terms: (“Behcet Syndrome”) AND (“HLA” OR “human leukocyte antigen”) AND (“polymorphism” OR “variant” OR “genotype” OR “allele”).

Selection criteria were as follows:

(1) HLA class I and II genes and any A, B, C, DRB1, DQB1, DQA1, and DRB1 alleles or molecules
(2) BS diagnosed following the clinical criteria set by the following:
  - International study group for Behcet's disease, Criteria for diagnosis of Behcet's disease, Lancet, 1990; 335: 1078-1080 [1]
  - International Team for the Revision of the International Criteria for Behcet's Disease. Evaluation of the International Criteria for Behcet's disease (ICBD) Clinical and Experimental Rheumatology. 2006; 24(supplement 42): p. S13 [13]
  - International Team for the Revision of the International Criteria for Behcet's Disease. Revision of the International Criteria for Behcet's Disease (ICBD) Clinical and Experimental Rheumatology. 2006; 24(supplement 42):S14–S15 [14]
  - International Team for the Revision of the International Criteria for Behçet's Disease (ITR-ICBD). The International Criteria for Behçet's Disease (ICBD): a collaborative study of 27 countries on the sensitivity and specificity of the new criteria. J Eur Acad Dermatol Venereol. 2014; 28(3):338-347 [15]
  - Behcet's Disease Research Committee of Japan. Behcet's disease guide to the diagnosis of Behcet's disease (1972) Japanese Journal of Ophthalmology. 1974; 18 : 291–294 [16]
  - Mizushima Y. Recent research into Behcet's disease in Japan. International Journal of Tissue Reactions. 1988; 10(2):59–65 [17]

### 2.3. Risk of Bias Assessment

Following a quality assessment tool for genetic data (Quality Assessment of Genetic Studies in Systematic Reviews, QUAGENS) [18], proposed by our multidisciplinary panel (statisticians, clinical epidemiologists, immunogeneticists, clinicians, and meta-analysts), three pairs of reviewers (one for the clinical criteria, one for laboratory issues, and one for methodology tools) working independently and with adequate reliability verified the following aspects:

1. Clinical data: the presence of spectrum disease biases, the possible enrollment of incident or prevalent cases, the inclusion of controls not selected from the same source population as the case-subjects, and the occurrence of differential participation in cases and controls
2. Laboratory issues: the misclassification of genotypes or serotypes (including the types and quality of samples, timing of collection, and the method used for HLA typing), the actual laboratory staff blinded to outcome, and the mention of quality controls
3. Methodological features: the possible population stratification, the presence of multiple testing and prestudy odds of true finding (it would be useful interpreting the results in the context of how many polymorphisms have been studied), and the assessment of HW equilibrium in controls

Each question was answered as “yes,” “no,” or “unclear.”

### 2.4. Data Extraction

After a critical reading of full-text articles, two investigators independently performed data extraction according to the selection criteria. The third participant was consulted for discussion to reach agreement concerning discrepancies. The following items were extracted from each study: first author's last name, publication date, country of origin, numbers of cases and controls, and typing method.

### 2.5. Data Synthesis and Meta-Analysis

STATA and Meta-DiSc was used for statistical analysis to perform the meta-analysis. Heterogeneity was checked by the chi-squared test and the *I*-squared statistics [19]. Statistical heterogeneity was defined by a *P* value < 0.10 for the chi-squared test and an *I* − squared statistics > 50%.

When there was no statistical evidence for heterogeneity in effect sizes, the fixed-effect model was used [20] to meta-analyze ORs or RRs in probands; when significant heterogeneity was identified, the random-effects model was used [21] to explore sources of significant heterogeneity. Also, a subgroup analysis stratified by ethnicity was performed.

## 3. Results

### 3.1. Study Characteristics and Quality Assessment

Following the search strategy, we identified 1141 articles, and after multiple rounds of selection, 68 articles were chosen for a full-text evaluation, of which 31 were included in the meta-analysis; 26 of them reported only the frequencies for the HLA − B∗51 allele [22–47].

**Figure 1:**
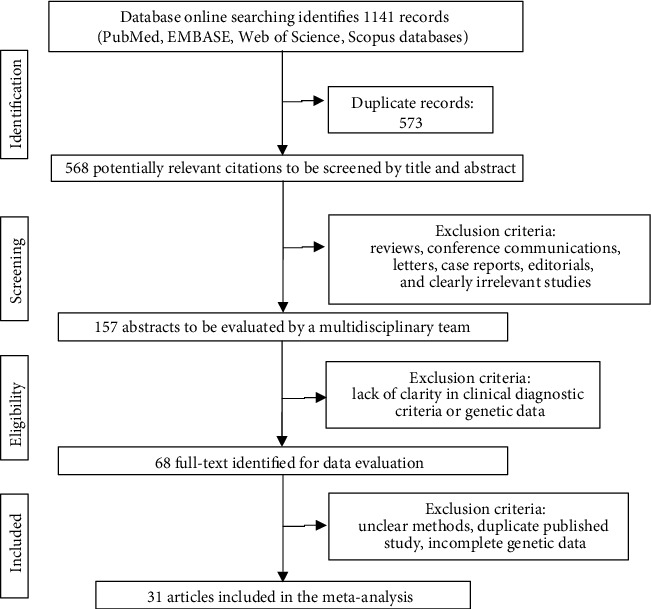
Flow diagram of the study following the PRISMA guidelines.

Figure 1 shows the flow diagram according to the PRISMA statement [12].

**Figure 2:**
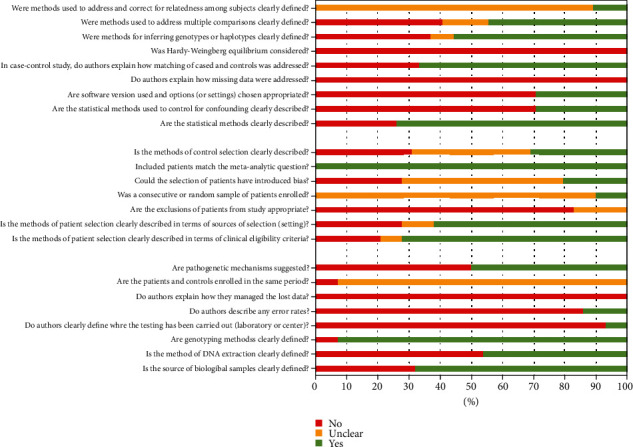
Quality assessment of studies. A series of questions were answered about laboratory methods, methodology, and clinical features. For each question, the answer is yes, no, or unclear.

The quality of studies in terms of laboratory method description, statistical methodology, and clinical features is depicted in Figure 2.

### 3.2. Meta-Analysis on the Association between Behcet Syndrome Susceptibility and HLA Alleles from Class I (HLA-A, B, and C) and II (HLA-DRB1, DQB1, and DPB1) Genes

We collected HLA genetic data from 31 case-control studies and retrieved frequencies for 24 HLA-A alleles and 3 alleles for HLA − A∗26 at four digits, 50 HLA-B alleles and 11 alleles for HLA − B∗51 at four digits, 15 HLA-C alleles, 16 HLA-DRB1 alleles, 6 HLA-DQB1 alleles, and 15 HLA-DPB1 alleles.

We evaluated the strength of the association between specific HLA alleles and the susceptibility to BS considering both predisposing and protective alleles. We meta-analysed only HLA allelic frequencies from at least three studies; therefore, we investigated only 21 alleles out of 140. Egger's regression test showed no evidence of publication bias (Egger's regression test *P* values > 0.1).

Table 1 lists the number of Behcet syndrome (BS) patients and the ethnically matched controls and the results of the meta-analyses carried out for each HLA allele to verify the correlation with susceptibility or protection to BS.

Going from 7.00 to 1.6 OR, we found 11 class I alleles conferring risk for BS: B∗51 : 08, B∗51, B∗51 : 01, B∗51 : 02, DQB1∗03, A∗26 : 01, Cw∗14, Cw∗15, Cw∗16, B∗15, and A∗26. On the contrary, going from 0.36 to 0.69, we found 11 class I alleles conferring a protective role to BS: B∗54, DQB1∗05, DRB1∗13, A∗33, B∗18, Cw∗03, B∗07, B∗52, B∗35, and Cw∗07 (Table 1).

**Figure 3:**
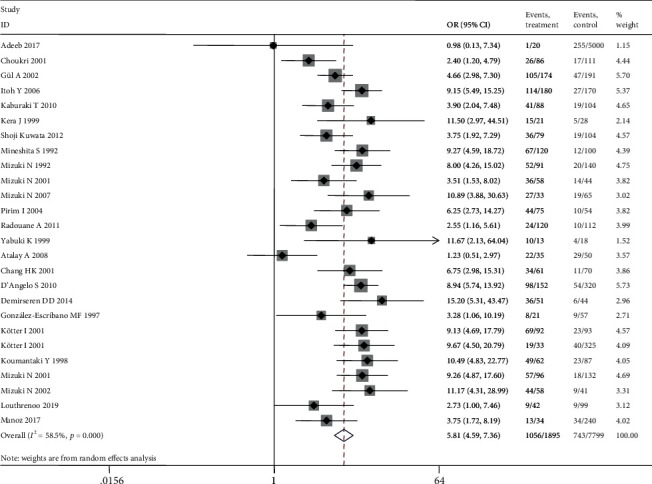
Forest plot from the meta-analysis for the HLA − B∗51 allele.

Figure 3 depicts the forest plot for the HLA − B∗51 allele, as it has been considered the most relevant genetic marker of the disease. All nationalities are listed on the left side for each included study.

### 3.3. Ethnicity-Specific Meta-Analysis

Overall, the studies included populations from Europe (Greece, Spain, Italy, Germany, and Ireland), Asia (Korea, China, China Han, and Thailand), Middle East (Israel, Saudi Arabia, and Iran), and Morocco (as no other North-African population was included).

Due to the higher frequency of BS among Japanese and Turkish people, we considered studies from Japanese and Turkish samples separately.

We collected a number of ethnical groups sufficient to conduct an ethnic-specific meta-analysis.  Table 2 lists the OR from the HLA alleles for each ethnic-subgroup.

## 4. Discussion

Since 1978, the autoinflammatory Behcet syndrome (BS) has been linked to the HLA (human leukocyte antigen) genetic system. Using serotype tests, BS was first linked to the HLA-B5 molecule; later, using molecular testing, BS was linked to the HLA-B51 allele, which is still the strongest genetic BS marker [43].

Going from the HLA-B gene to the telomere region of chromosome 6, we have HLA-C and HLA-A genes, while going from the HLA-B gene to the centromere region, we have HLA class III genes (not included in this study) and HLA class II genes, such as HLA-DRB1, HLA-DQB1, and HLA-DPB1.

Here, for the first time, we meta-analysed the HLA class I and II genes involved in BS susceptibility in all ethnic worldwide groups, collecting HLA genetic data from 31 case-control studies and retrieved allelic frequencies for the HLA-A, HLA-B, HLA-C, HLA-DRB1, HLA-DQB1, and HLA-DPB1 genes.

Our data confirmed the HLA-B51 allele as the genetic HLA marker mostly associated with BS development all over the world among all different ethnic samples (OR 5.81, *P* < 0.0001) (Figure 3).

Notably, considering high-resolution two-digit analysis of the HLA-B51 allele, we observed that the HLA − B∗51 : 08 variant showed the highest OR associated with BS (OR = 7.00, *P* < 0.001) (Table 1), followed by the two-digit HLA − B∗51 : 01 and HLA − B∗51 : 02 alleles (Table 1). Moreover, in our set of Europeans, the HLA − B∗51 : 08 variant showed a 11.25 OR (Table 2).

Our results are in line with the work by Guasp and colleagues where high-resolution HLA − B∗51 alleles were associated with epistasis with Hap10, a low-activity variant of ERAP1 (endoplasmic reticulum aminopeptidase 1), although its pathogenic role in BS is still unclear. In particular, the authors studied the effects of Hap10 on the HLA − B∗51 peptidome aiming at distinguishing the different effects of this epistasis with high-resolution HLA − B∗51 polymorphisms in BS [47]. The HLA − B∗51 : 08 BS-associated peptidome expressed in a Hap10-positive cell line was compared with the HLA − B∗51 : 01 peptidome from cells expressing more active ERAP1 variants. The authors estimated that peptide-binding affinity and the HLA − B∗51 : 08 peptidome generated longer peptides. They concluded that the BS-associated Hap10 haplotype induces changes in the repertoire of peptides presented to HLA − B∗51 altering its antigen-presenting specificity and generating a lower affinity peptidome [45].

In our analysis we also observed an association with BS with the HLA − A∗26 : 01 allele (HLA class I), the HLA − Cw∗14, HLA − Cw∗15, and HLA − Cw∗16 alleles (HLA class I) and with HLA − DQB1∗03 allele (HLA class II) (Table 1).

As to HLA − A∗26 risk allele, our real-world data are a confirmation of the results imputed by Ombrello and colleagues, who inferred in 2014 the independent role of HLA-B51 and HLA-A26 in BS susceptibility by imputed MHC-region SNPs and also found HLA − B∗15 to be an independent BS risk [48]. Moreover, we observed in a sample of 600 healthy subjects (personal unpublished data) that the HLA − A∗26;B∗51 haplotype was not the most frequent among HLA-A;B haplotypes (seventh place), thus further confirming with our real-world data the results by Ombrello and colleagues [48].

Regarding the association between BS and HLA-C alleles, Hughes and colleagues genotyped 8572 SNPs to infer classical HLA alleles in the HLA-A, HLA-B, HLA-C, HLA-DQA1, HLA-DQB1, and HLA-DRB1 genes from 2 ancestry groups, and they imputed data suggesting a robust HLA − B∗51 association with BS and an additional independent genetic association with HLA − Cw∗16 : 02 [49]. In agreement with imputed data by Hughes and colleagues, in our sample of 600 healthy Caucasian subjects (personal unpublished data), we observed that the HLA − B∗51;Cw∗15 and HLA − B∗51;Cw∗14 haplotypes were the second and third most frequent haplotypes, while the HLA − B∗51;Cw∗16 haplotype was the sixth place, thus supporting an independent role of HLA − B∗51 and HLA − Cw∗16 in BS.

Finally, we found that the HLA − DQB1∗03 was a BS risk allele (Table 1). To take into account all possible bias due to some linkage, we also observed in the same sample of 600 healthy subjects (personal unpublished data) that the frequency of the B∗51;DQB1∗03 haplotype was the second place.

Piga and colleagues found the HLA-A2; Cw2; B∗5101; DRB1∗11; DQA1∗05; DQB1∗03 haplotype in a subset of BS patients; however, the authors also found that the HLA-A2; Cw2; B∗5101; DRB1∗04; DQA1∗03; DQB1∗03 haplotype was not associated with BS, thus highlighting the importance of studying extended HLA haplotypes rather than single alleles [50].

Finally, we considered populations from Europe (Greece, Spain, Italy, Germany, and Ireland), Asia (Korea, China, China Han, and Thailand), Middle-East (Israel, Saudi Arabia, and Iran), and Morocco (as no other North-African population was included), and we separately considered studies from Japanese and Turkish samples due to their higher frequency of BS.

In our study, the most remarkable result was that the most frequent HLA − B∗51 two-digit alleles associated with BS were different in different populations: in Europe, the HLA − B∗51 : 08 (OR 11.25 C.I. 4.9-26), in Turkey the HLA − B∗51 : 01 (OR 5.98 C.I. 3.7-9.8), and in Japan the HLA − B∗51 : 02 (OR 5.39 C.I. 0.6-47) (Table 2).

On the whole, HLA − B∗51 is no more the only flag tagging a genetic marker to BS susceptibility; in fact, we observed that HLA-A and HLA-C variants also play an independent role in BS risk.

Beyond the distribution of HLA variants related to different ethnicities, we suggest that a further study ought to be focused on the correlation between these HLA − B∗51 two-digit variants (in particular HLA − B∗51 : 08) and clinical signs.

## 5. Conclusion

Despite remarkable different results on the distribution of the two-digit HLA − B∗51 alleles associated with BS among populations, unfortunately, we could not find sufficient data on the association between HLA alleles and different clinical features. This comparison should be a further goal in order to find also clinically relevant differences in treatment response.

## Figures and Tables

**Table 1:** HLA alleles involved in susceptibility/protection in Behcet syndrome (BS). Number of studies, number of BS patients and the ethnically matched controls, OR, and *P* values for each HLA allele included in the meta-analysis.

HLA	OR	*P*	*N* of BD	*N* of CTR	*N* of studies
B∗51 : 08	7.00	<0.0001	503	962	8
B∗51	5.81	<0.0001	1895	7799	26
B∗51 : 01	5.54	<0.0001	988	1571	13
B∗51 : 02	3.14	0.008	544	625	8
DQB1∗03	2.60	<0.0001	153	399	4
A∗26 : 01	2.48	<0.0001	432	1705	4
Cw∗14	2.35	0.001	279	260	4
Cw∗15	2.34	0.001	279	260	4
Cw∗16	2.23	0.014	279	260	4
B∗15	1.79	0.004	433	5479	5
A∗26	1.70	<0.0001	523	1781	7
B∗35	0.69	0.008	691	5763	10
Cw∗07	0.69	0.035	370	400	5
B∗52	0.58	0.007	715	855	10
B∗07	0.55	0.007	658	5698	9
Cw∗03	0.55	0.002	237	302	3
A∗33	0.53	<0.0001	661	1852	6
B∗18	0.53	0.017	580	623	8
DRB1∗13	0.51	0.015	267	554	6
B∗54	0.36	<0.0001	445	501	3
DQB1∗05	0.36	0.002	121	89	3

**Table 2:** Meta-analysis for ethnic subgroups. Values are odds ratio (OR) and confidence interval (CI) in parentheses.

	Overall	Asia	Europe	Middle East	Morocco	Turkey	Japan
A∗26 : 01	2.48 (1.8-3.5)	1.89 (1.2-2.8)					3.50 (2.1-5.8)
B∗15	1.79 (1.2-2.7)		2.53 (0.8-7.6)		1.92 (1.0-3.5)	1.51 (0.8-2.7)	
B∗51	6.07 (4.8-7.8)	5.99 (3.1-12)	8.22 (5.6-12)	5.03 (1.7-15)	2.46 (1.5-4.1)	5.97 (3.2-11)	6.44 (4.3-9.6)
B∗51 : 01	5.57 (4.5-6.8)		5.16 (3.7-7.2)			5.98 (3.7-9.8)	6.12 (4.5-8.3)
B∗51 : 02	3.14 (1.3-7.3)					2.91 (1.0-8.2)	5.39 (0.6-47)
B∗51 : 08	7.00 (3.8-13)		11.25 (4.9-26)			3.96 (1.6-9.9)	
B∗52	0.58 (0.4-0.9)	0.69 (0.2-2.7)	0.25 (0.1-5.4)	1.01 (0.2-4.8)	0.93 (0.2-4.7)	0.63 (0.3-1.5)	0.51 (0.3-0.9)
B∗54	0.36 (0.2-0.6)					0.36 (0.1-9.0)	0.36 (0.2-0.6)

## Data Availability

Data is available at http://www.crd.york.ac.uk/PROSPEROCRD42019130390
